# Home Telemonitoring of Arterial Hypertension With Antihypertensive Treatment Titration: Protocol for a Randomized Controlled Prospective Trial (HOROSCOPE Study)

**DOI:** 10.2196/26184

**Published:** 2022-03-01

**Authors:** Sonia Ben Hafaiedh, Yosra Ben Daya, Amina Hadjer Radoui, Mohamed Bouchoucha, Rabie Razgallah, Semir Nouira

**Affiliations:** 1 Medical Department MédiS Laboratories Tunis Tunisia; 2 Dacima Tunis Tunisia; 3 Emergency Department and Laboratory Research (LR12SP18) Fattouma Bourguiba University Hospital Monastir Tunisia

**Keywords:** telemonitoring, arterial hypertension, primary care, ambulatory blood pressure monitoring, randomized controlled trial

## Abstract

**Background:**

Despite the availability of effective treatment, the control of hypertension remains insufficient. Telemonitoring in the management of hypertension would be an effective way to improve blood pressure control.

**Objective:**

The aim of our study will be to evaluate the effects of telemonitoring with antihypertensive treatment titration on blood pressure control in Tunisian patients with hypertension.

**Methods:**

Our trial will be a prospective, rater-blinded randomized controlled trial carried out with primary care physicians in the Sahel region of Tunisia. Patients will be eligible for enrollment if they are aged over 35 years, are newly diagnosed with hypertension, or are known to be poorly controlled on antihypertensive therapy. Participants will be randomly assigned in a 1:1 ratio to the telemonitoring arm or usual care arm. The telemonitoring arm will involve a weekly telephone call for the collection of the home blood pressure measurements, therapeutic education, and treatment compliance assessment as well as a monthly call for treatment titration and a side effect check. Randomization will be done via the use of an interactive web responsive system, and patients will be stratified by investigation center. Neither participants nor investigators will be masked to the group assignments. The primary outcome will be the change in mean 24-hour systolic blood pressure from baseline to the 6-month follow-up in the 2 groups. All randomized patients who attend the follow-up visit at 6 months and have no missing data for the primary outcome will be included in the analysis.

**Results:**

Recruitment to the trial started in July 2020. The study was initiated with 17 primary care physicians. We expect the inclusion period to last for approximately 6 months. We expect to complete data collection by the end of 2021 and plan to disseminate the results subsequently.

**Conclusions:**

The HOROSCOPE (Home Telemonitoring of Arterial Hypertension With Antihypertensive Treatment Titration: Randomized Controlled Prospective Trial) study will provide important new evidence that could shed some light on the feasibility and impact of telemonitoring and self-monitoring in a Tunisian population of patients with hypertension who consult primary care physicians.

**Trial Registration:**

ClinicalTrials.gov NCT04607239; https://clinicaltrials.gov/ct2/show/NCT04607239

**International Registered Report Identifier (IRRID):**

DERR1-10.2196/26184

## Introduction

### Background

Hypertension is one of the main risk factors for cardiovascular disease and the leading cause for global morbidity and mortality [1,2]. Despite the availability of effective treatment, the control of hypertension remains insufficient [3]. It has been suggested that self-monitoring in the management of hypertension is an effective method of improving blood pressure control [4,5]. Nevertheless, isolated self-monitoring has not been associated with better blood pressure control; it would be more beneficial if it was used in combination with other interventions, such as integrating primary care physicians in a telemonitoring approach. Despite conflicting evidence, a Scottish study involving general practitioners reported a significant reduction in blood pressure over a period of 6 months [6]. Furthermore, telemonitoring could help physicians with antihypertensive treatment titration [7], as shown in another trial (TASMINH2 [Telemonitoring and/or Self-monitoring of Blood Pressure in Hypertension 2]), which demonstrated that the telemonitoring with home titration group had lower blood pressure than that of the usual care (control) group after 12 months [8]. Moreover, a recent study (TASMINH4 [Telemonitoring and/or Self-monitoring of Blood Pressure in Hypertension 4]) aimed to determine whether telemonitoring resulted in a lower blood pressure by comparing it to usual care (control) and self-monitoring only [9]. The trial was a parallel randomized controlled trial that was conducted with 142 general practitioners in the United Kingdom and included patients who were hypertensive, aged over 35 years, had a blood pressure of >140/90 mm Hg, and were willing to self-monitor their blood pressure. A total of 1182 patients were randomly assigned to blood pressure self-monitoring (self-monitoring group: n=395), blood pressure self-monitoring with telemonitoring (telemonitoring group: n=393), or usual care (control group: n=394). After 12 months, patients’ systolic blood pressure (SBP) was lower in both the self-monitoring and telemonitoring groups compared to that of the usual care group (mean 137, SD 16.7 mm Hg and mean 136, SD 16.1 mm Hg vs mean 140.4, SD 16.5 mm Hg, respectively). No statistically significant differences between the self-monitoring and telemonitoring groups were recorded. This study showed that self-monitoring, with or without telemonitoring, can be used to guide the titration of antihypertensive medicines in primary care. The observed drops in blood pressure in this study indicated an approximate 20% and 10% reduction in the risk of stroke and coronary heart disease, respectively [9]. The results of this study deserve to be confirmed, especially since this study’s approach can be economically interesting [10] in the context of a resource-limited country with failing health economics. It would be of great use to evaluate this approach with general practitioners in Tunisia—a country in which the prevalence of hypertension in urban areas is significantly greater than that in rural areas (*P*<.001) and only 38.1% of patients with hypertension are aware of their hypertension [11].

### Research Questions

The aim of the HOROSCOPE (Home Telemonitoring of Arterial Hypertension With Antihypertensive Treatment Titration: Randomized Controlled Prospective Trial) study is to demonstrate the impact of telemonitoring on blood pressure control among patients with hypertension.

The trial will address the following three main research questions: (1) does telemonitoring improve treatment compliance and blood pressure control in newly diagnosed or poorly controlled patients with hypertension, (2) does telemonitoring improve patients’ quality of life, and (3) does telemonitoring reduce the incidence of cardiovascular complications?

## Methods

### Study Design

Our trial will be a multicentric, rater-blinded, prospective randomized controlled trial. The allocation ratio between the intervention and control group will be 1:1. Randomization will be done via the use of an interactive web responsive system, and patients will be stratified by investigation center. Neither participants nor investigators will be masked to the group assignments. However, the cardiologist, who will interpret the results of the primary outcome, will be blinded.

The study is expected to last for an overall duration of 12 months (from the first patient who is enrolled to the last patient who completes the trial). The recruitment period will last for 6 months, and the follow-up interval will be 6 months.

### Participants

Patients with hypertension will be recruited from consultations with primary care general practitioners in the central region of Tunisia. The inclusion and exclusion criteria are outlined in Textbox 1.

**Inclusion and exclusion criteria for the HOROSCOPE (Home Telemonitoring of Arterial Hypertension With Antihypertensive Treatment Titration: Randomized Controlled Prospective Trial) study.**

**Inclusion criteria**

- Aged ≥35 years old
- Newly diagnosed hypertension
- Poorly controlled hypertension
  One single blood pressure value outside the expected range will not be taken as a definitive indicator of clinical deterioration
  Blood pressure should be >140/90 mm Hg at screening for at least 2 visits to allow the inclusion of a patient

**Exclusion criteria**

- Orthostatic hypotension
- Chronic renal failure (serum creatinine level: >200 µmol/L)
- Acute coronary syndrome, coronary revascularization, or stroke within the past 3 months
- Known secondary causes of hypertension
- Pregnancy
- New York Heart Association Class III or IV heart failure or left ventricular ejection fraction of <30%
- Dementia or any other cause that could prevent the implementation of remote monitoring (telemonitoring)

### Recruitment, Screening, and Informed Consent Procedures

Participants will be recruited directly from primary health care centers by general practitioners. Participants will be included during their visit to the outpatient clinic after the verification of their eligibility criteria. When patients meet the eligibility criteria, the investigator will provide them with a study information sheet. Patients showing interest in taking part in the study will be included.

**Figure 1:**
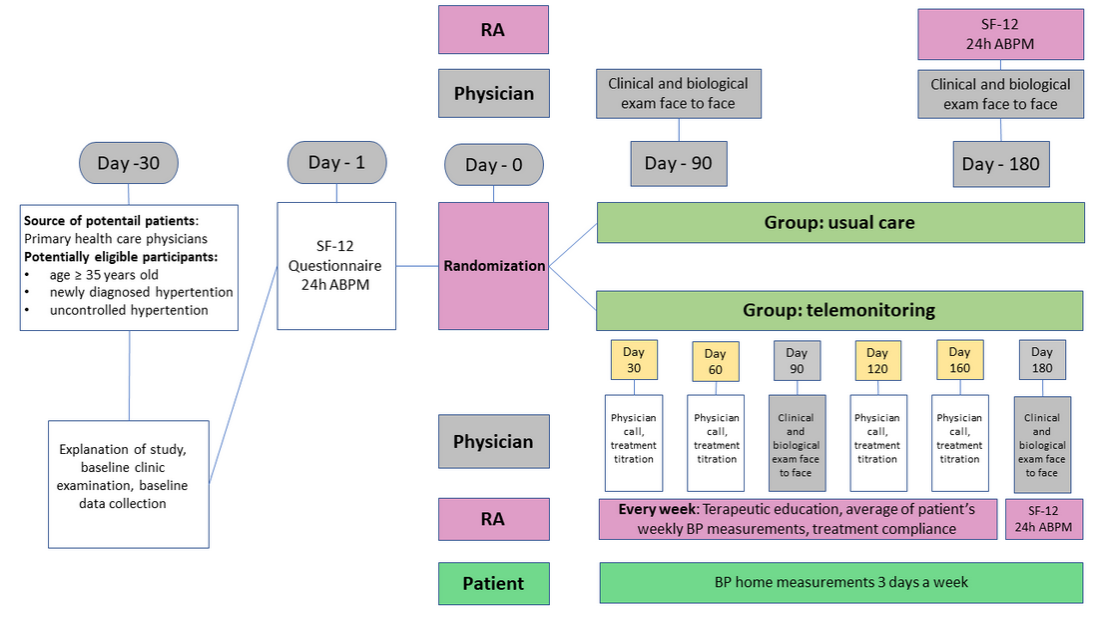
Participant flow throughout the study. ABPM: ambulatory blood pressure monitoring; BP: blood pressure; CRA: clinical research associate; SF-12: 12-Item Short Form Survey.

Material compensation will not be offered for participating in the study. Potential participants will be informed about the scientific benefits of their participation and will not be offered any other incentives. In order to obtain informed consent, the investigator will inform the participants about the possible benefits and potential side effects of participating in this study as well as the study duration. Patients will also be informed that their participation will be strictly voluntary and that they can withdraw from the study at any time without stating any reason. They will also be informed that withdrawing from the study will not affect subsequent medical assistance and treatment. Participants will be advised that their personal data will be used by other authorized health care professionals other than their treating physicians as part of the clinical trial. They will be given sufficient time to reflect extensively on their participation decision. The informed consent form will be signed and dated by the investigator following the signature of the participant. The consent form will be retained as part of the study records. A signed copy will be provided to each study participant. Overall patient flow throughout the study is depicted in Figure 1.

### Sample Size

A sample size of 199 subjects per group is required for 80% power, assuming a mean difference in the reduction of SBP of at least 5 mm Hg between the two study groups and an SD of 15 mm Hg. Considering a patient dropout ratio of 15%, the study will need to recruit 230 subjects in each group.

### Baseline and Follow-up Clinic Examinations

Patients will undergo an initial baseline clinic examination, during which clinical measurements, demographic data, and past medical histories will be collected and electrocardiogram exams and biological examinations will be conducted.

A validated blood pressure monitor (ROSSMAX X3; Rossmax Swiss GmbH) will be used for blood pressure measurements. After 1 month, a satisfaction questionnaire—the Medical Outcomes Study Short Form-12 questionnaire [12]—will be completed by participants, so that we can assess their quality of life.

Twenty-four–hour ambulatory blood pressure monitoring (ABPM) will be performed for all included patients to analyze their blood pressure profiles through the use of a Holter monitor (Contec ABPM 50; Contec Medical Systems). Ambulatory blood pressure measurements will be scheduled to acquire a blood pressure reading every 30 minutes from 6 AM to 10 PM and every 60 minutes from 10 PM to 6 AM. The cuff will be placed on participants’ nondominant arms. Of the 48 possible measurements, at least 32 (67%) must be available. Otherwise, ABPM will be repeated. The Holter monitor must not get wet (eg, via a swimming pool, bath, or shower), and usual activities must be maintained. After installing the Holter monitor, study participants can return to their usual activities. They will be invited to return to the clinic 24 hours later, so that we can collect the data from their monitors. The results will be interpreted by a cardiologist.

Randomization will take place 1 month after participants’ inclusion, that is, after the collection of at least 32 interpretable ABPM measurements. All patients will be asked to attend 2 follow-up clinics within 6 months. The clinical follow-up data to be collected are detailed in Table 1.

**Table 1:** Timetable for patient data collection throughout the trial.

Collected data	Baseline (inclusion visit)	After 29 days (1 day prior to the randomization visit)	After 3 months (follow-up visit 1)	After 6 months (follow-up visit 2)
Sociodemographics	✓			
Past medical history	✓			
Clinical examination^a^	✓	✓	✓	✓
Biological tests^b^	✓		✓	✓
ECG^c^	✓	✓		✓
SF-12^d^		✓		✓
ABPM^e^		✓		✓
Treatment compliance		✓	✓	✓
Side effects		✓	✓	✓

^a^The clinical examination will mainly include the assessment of blood pressure (systolic and diastolic blood pressure), BMI, and heart failure New York Heart Association stage.

^b^Biological tests will include those for serum creatinine, glycemia, lipid profiles, and brain natriuretic peptides.

^c^ECG: electrocardiogram.

^d^SF-12: 12-Item Short Form Survey.

^e^ABPM: ambulatory blood pressure monitoring.

### Randomization

Patients who meet the eligibility criteria will be randomly assigned to either of the study groups in a 1:1 ratio via the use of an interactive web responsive system.

The study groups are as follows:

- Usual care group (control group): patients in this group will receive their antihypertensive treatment according to the usual approach during each consultation appointment made with the attending physicians.
- Telemonitoring group (intervention group): patients in this group will receive their usual care along with at-home self-monitoring, telemonitoring, and the titration of antihypertensive treatment.

### Intervention

#### Control Group

Participants randomized to the control group will receive usual care (ie, physician visits every 3-6 months and therapy according to standard treatment guidelines). In contrast to the intervention group, control group participants will not receive phone calls for therapeutic education, a treatment compliance assessment, treatment titration, or a side effect check. In cases of any worsening symptoms, patients will have to contact their general physician individually and independently.

#### Intervention Group

Participants randomized to the intervention group will receive therapeutic education, including information on diet, sleep, physical activity, and other lifestyle advice, from the research associate. Patients will also be provided with a validated blood pressure monitor (ROSSMAX X3), and they will be taught how to use the blood pressure monitor at home. Patients will be asked to measure their blood pressure 3 times in the morning and 3 times in the evening for 3 days per week.

Once per week, the research associate will call the patients to check their treatment compliance and verbally collect the blood pressure measurements. Compliance evaluation will be conducted by asking the patients whether they have been taking their medication correctly every day on time for the past week. Patients will be asked to keep their empty treatment boxes. The remaining pills will be counted, and patient compliance will be assessed. The research associate will also remind patients about lifestyle and dietary measures. Subsequently, the research assistant will fill in the data entry fields in the electronic platform, which can be checked and validated by the investigators. Patients will be contacted every month via telephone by their attending physician for 6 months to titrate the antihypertensive treatment according to the data collected by the research associate. At day 90 and day 180, the medical visit will be conducted face-to-face. For the titration of antihypertensive medicines, the decision criterion will be the percentage of home blood pressure readings that meet the target. If at least 75% (375/500) of the readings since the last visit meet the blood pressure target, no changes in treatment will be considered. If less than 75% (375/500) of the readings meet the target, we will recommend readjusting the treatment. During the telephone follow-up calls, physicians will encourage participants to adhere to the treatment. Regardless of blood pressure control, if patients experience side effects, the involved antihypertensive drug will be stopped or changed, or its dose will be reduced.

### Target Blood Pressure

The target blood pressure will be based on the 2018 European Society of Cardiology and European Society of Hypertension Guidelines [13].

The home blood pressure target will be ≤135/85 mm Hg, and the general practitioners’ office blood pressure target will be ≤140/90 mm Hg. The mean ambulatory blood pressure targets will be ≤135/85 mm Hg at daytime, ≤120/70 mm Hg at nighttime, and ≤130/80 mm Hg for 24 hours.

### Statistical Analysis

Categorical variables will be expressed as frequencies and percentages, while continuous variables will be expressed as means with SDs. An analysis of variance will be performed to compare the two study groups in terms of the primary end point. The normality of continuous data will be assessed with the Shapiro-Wilk test. Furthermore, we will use a general linear model for repeated measures to compare the marginal means of Gaussian quantitative dependent variables before and after the intervention (a Bonferroni test will be considered). A nonparametric test (the Friedman test) will be used for the comparison of non-Gaussian quantitative variables before and after intervention. The Cochran Q test will be performed to compare binary dependent variables before and after intervention. Subgroup analyses will be performed by age (<65 years and ≥65 years), sex, and the severity of hypertension. Differences between continuous variables will be evaluated with a 2-tailed Student *t* test or by its correction, as appropriate. The differences among the categorical variables will be evaluated with the Pearson chi-square test. All tests will be considered significant at the cutoff value of *P*<.05.

Data will be collected via an electronic data capture system (Dacima Clinical Suite; Dacima Software Inc) that is compliant with international data security requirements (ie, those of the US Food and Drug Administration [FDA] Title 21 of the Code of Federal Regulation [CFR], part 11; Health Insurance Portability and Accountability Act [HIPAA]; International Council for Harmonisation of Technical Requirements for Pharmaceuticals for Human Use [ICH]; and General Data Protection Regulation [GDPR]). Dacima Clinical Suite is authorized by the local body of personal data protection. The electronic case report form will be used to perform quality checks on the data (checks on simple controls [eg, range check], eligibility criteria, and complex controls) to increase the accuracy of the data. Double data entry will not be scheduled, as data will be directly captured in the electronic data system. Investigators and research assistants will use monitoring data tools as an audit trail for tracking the data modification log, data queries, and electronic signatures. Research assistants will use source document verification tools to ensure the accuracy of the collected data. Automatic alerts will be implemented to notify the responsible investigator about any data or protocol violations.

Participants who do not have data on the primary outcome at the 6-month follow-up assessment will be labeled as those who are missing data for the primary outcome at this time point. A further sensitivity analysis will include participants with 1 missing measurement. The frequency (percentage) of losses to follow-up (defaulters and withdrawals) after 6 months will be reported by randomized group and compared between the two groups. The availability of the outcome data for the primary and secondary outcomes will be summarized for the two randomized groups. The mixed-effects model implicitly accounts for data missing at random; however, the missing data mechanism will be explored. A logistic regression model will be used to explore any associations between baseline characteristics and the availability of data on the primary outcome. Any changes to the assumptions made in the primary analysis (ie, data missing at random) will be considered in a sensitivity analysis.

### Outcomes

#### Primary Outcome

Our primary outcome will be the percentage of patients who exhibit a change of 10 mm Hg in average 24-hour SBP from baseline to the 6-month follow-up.

#### Secondary Outcomes

The following will be our secondary outcomes:

- The percentage of patients who exhibit a change of 5 mm Hg in average 24-hour diastolic blood pressure from baseline to the 6-month follow-up
- The percentage of patients who exhibit a change of 10 mm Hg and 5 mm Hg in average 24-hour blood pressure from baseline to the 6-month follow-up
- The change in blood pressure load (Textbox 2) percentages from baseline to the 6-month follow-up
- The change in dipping (Textbox 2) percentages from baseline to the 6-month follow-up
- The percentage of patients taking more than 80% (400/500) of the antihypertensive medications at the 6-month follow-up
- The percentage of patients with changes in mean 12-Item Short Form Survey scores from baseline to the 6-month follow-up
- Clinical outcomes, including hospitalization rates and emergency room admissions related to high blood pressure, adverse effects, or other reasons

**Blood pressure load and dipping definitions.**

**Definitions**

- Blood pressure load: the percentage of abnormally elevated blood pressure readings, which is usually provided in the ambulatory blood pressure monitoring report. Normal values should be <40% [14,15].
- Dipping: the difference between the mean systolic pressure during the day and mean systolic pressure during the night. This is expressed as a percentage of the daytime mean, and the accepted normal ranges between 10% and 20% [16].

### Ethics and Dissemination

Full ethical approval for the trial was obtained from the local ethics committee of Monastir University of Medicine (reference number: IORG 0009738 N° 50/098060279). The study is registered on the ClinicalTrials.gov registry under the identifier NCT04607239.

The electronic data capture system that was provided to the study is compliant with confidentiality requirements (ie, those of the US FDA Title 21 of the CFR, part 11; HIPAA, ICH; and GDPR), and local regulatory authorization was obtained before starting the project. Personal data that are not relevant to the study will not be collected. Other personal data will be secured by obtaining specific rights for their use in follow-up monitoring only. Investigators and research associates will sign a confidentiality agreement before starting the project.

The final data set will be extracted from the electronic data capture system along with anonymized personal data. The primary investigator will be responsible for access to the final data set for medical writing purposes. The Contract Research Organization team of Dacima is authorized to access the final data set to program statistical analyses and provide the final results of the project according to the statistical analysis plan. The primary investigator will be responsible for reviewing the data analysis and validating the final clinical study report.

## Results

Recruitment to the trial started in July 2020. The data collection period is expected to last for approximately 12 months (from the first patient who is enrolled to the last patient who completes the trial). The length of the study per patient will be 6 months. We expect to complete data collection by the end of 2021 and plan to disseminate the results subsequently.

## Discussion

### Study Contributions

This paper describes the protocol for the HOROSCOPE study—a randomized controlled trial that will assess the impact of telemonitoring on the control of blood pressure. To our knowledge, this will be the first Tunisian study to compare telemonitoring to usual care in terms of the control of blood pressure. If telemonitoring is found to be successful, we will extend the study to other investigation centers throughout the Tunisian territory. We can, in the future, develop connected electronic devices that can send the results of blood pressure measurements directly to doctors.

### Strengths and Limitations

Randomized controlled trials are considered the “gold standard for evaluating the efficacy of different interventions in clinical research and constitute evidence for medical treatment” [17]. By conducting such a trial and ensuring internal validity, we will maximize the robustness of our study.

A possible limitation is that enrolled patients may greatly vary in terms of their clinical profiles. In addition, subjects may feel overloaded by the daily measurements and questionnaires that need to be answered, which could result in dropouts. Another possible limitation is that the study population may not represent the overall Tunisian population.

## Abbreviations

ABPM: ambulatory blood pressure monitoring
CFR: Code of Federal Regulation
FDA: Food and Drug Administration
GDPR: General Data Protection Regulation
HIPAA: Health Insurance Portability and Accountability Act
HOROSCOPE: Home Telemonitoring of Arterial Hypertension With Antihypertensive Treatment Titration: Randomized Controlled Prospective Trial
ICH: International Council for Harmonisation of Technical Requirements for Pharmaceuticals for Human Use
SBP: systolic blood pressure
TASMINH2: Telemonitoring and/or Self-monitoring of Blood Pressure in Hypertension 2
TASMINH4: Telemonitoring and/or Self-monitoring of Blood Pressure in Hypertension 4
